# Walking football for people with chronic breathlessness: A mixed-methods proof of concept study

**DOI:** 10.1177/14799731261489273

**Published:** 2026-09-21

**Authors:** Callum Bradford, Kirsti J. Loughran, Benjamin Harrison, Grant McGeechan, Denis Martin, Sophie Suri, Noelle Robertson, Gaynor Williams, Tim Rapley, Samantha L. Harrison

**Affiliations:** 1 School of Health and Life Sciences, 5462 Teesside University, Middlesbrough, UK; 2 NIHR Applied Research Collaboration (ARC) North East and North Cumbia (NENC), Newcastle upon Tyne, UK; 3 School of Social Science Humanities and Law, Teesside University, Middlesbrough, UK; 4 School of Psychology and Vision Sciences, 4488 University of Leicester, Leicester, UK; 5 Breathe Easy Darlington, Darlington, UK; 6 School of Communities and Education, 5995 Northumbria University, Newcastle-upon Tyne, UK

**Keywords:** chronic breathlessness, exercise-maintenance, pulmonary rehabilitation, walking football, mixed-methods

## Abstract

**Introduction:**

Pulmonary rehabilitation (PR) provides substantial benefits, but these are rarely sustained. Walking football (WF), with its slower pace and widespread availability, may offer an exercise-maintenance option. This proof-of-concept study aimed to understand the potential of WF as an exercise-maintenance intervention following PR.

**Methods:**

A mixed-methods, prospective observational design was used. Patients completing PR at two NHS trusts were invited to attend weekly 2-hour WF sessions for 6 weeks. Feasibility outcomes included recruitment, adherence, and safety. An ethnographic approach observed participant experiences and engagement. Health outcomes were assessed at baseline and post-intervention, and the intensity of a single WF session was analysed.

**Results:**

160 individuals were approached; 36 expressed interest and 6 enrolled (5 male; mean age 71 years). Travel difficulties were the most common reason for declining. Five participants attended at least 4 sessions, and all continued WF after the study. One minor, unrelated adverse event occurred. Qualitative analysis of 18-hours of observations and 6 interviews generated 6 themes. The most notable finding highlighted the importance of WF sessions being exclusive to people with breathlessness. At least 3 participants achieved the minimal clinically important difference in functional exercise capacity, strength, health-related quality of life, and symptom burden. During a single session, group mean heart rate reached 66% of estimated HRmax, with a mean distance of 3.23 km covered.

**Conclusions:**

WF appears to be a promising, engaging, and potentially sustainable exercise-maintenance option following PR, though travel barriers limited uptake. Future interventions should prioritise accessible locations and consider transport support.

## Introduction

Chronic breathlessness is a persistent, debilitating symptom and a common consequence of chronic lung diseases, most commonly Chronic Obstructive Pulmonary Disease (COPD). COPD is a highly prevalent and costly progressive lung condition. 1.2 million people are diagnosed in the UK and costs to the healthcare service exceed £810-930million annually.^1^ By 2030 COPD is projected to be the 3^rd^ leading cause of death worldwide.^2^ COPD is characterised by breathlessness but multimorbidity is common (51-96%),^3^ resulting in a loss of function and leading to a gradual decline in people’s ability to manage activities of daily living.^4^

Pulmonary rehabilitation (PR), an exercise and education programme, is recommended for individuals with breathless symptoms.^5,6^ The evidence for PR improving exercise capacity and quality of life is unequivocal,^5,6^ with the NHS long-term plan targeting the expansion of PR services.^7^ However, any benefits gained often subside within 3-6 months of programme completion, as individuals struggle to sustain exercise levels.^8^

Walking football is a modified form of football in which running is not permitted, at least one foot is required to remain in contact with the ground at all times. The game is typically played on a smaller pitch with adapted rules to reduce physical demands while retaining the social, competitive, and enjoyable aspects of football. Walking football offers a potential solution for exercise maintenance, with its reduced pace and ubiquity, it attracts individuals with a variety of health conditions, of varying age (33-81y), and from both sexes (55% male: 44% female).^9^ However, in its current form walking football may not be suitable for people with chronic breathlessness, the majority of whom are frail (58%).^10^ During patient and public involvement and engagement (PPIE) activities, two members with chronic lung disease reported attending walking sport sessions aimed at older adults and either stopped going because the pace was too quick or being asked to leave because they couldn’t keep up with the pace. Therefore, we co-designed a walking football intervention for people with chronic breathlessness (manuscript submitted for publication), and the aim of this study was to evaluate its potential as an exercise maintenance intervention following PR.

## Methods

### Study design and approval

A mixed-methods, prospective, observational, proof-of-concept study design was used. The specific objectives were to: 1. assess the feasibility of delivering walking football post-PR, 2. explore participants’ experience and engagement, 3. collect early indicators of physical benefits. Ethics approval was obtained from Teesside University Health Research Ethics sub-committee and North East - Tyne & Wear South Research Ethics Committee (REC reference 22/NE/0229) and written informed consent was obtained from all participants. The study was registered on clinicaltrials.gov (Clin Trials ID: NCT05575102).

### Participant recruitment

Individuals with chronic breathlessness enrolled in PR services at two NHS trusts were invited to participate between February to August 2023. Participants who met the following criteria were included: Chronic breathlessness due to an underlying chronic respiratory condition, stable condition (six weeks clear of exacerbation), scheduled to complete PR between January and July 2023, ≥35y of age, able to walk 10m unassisted and able to communicate with good verbal English or use adaptive equipment to communicate. Individuals with Long Covid were excluded because the condition’s health effects remain insufficiently defined to ensure safety or avoid potentially confounding influences on outcomes. Those considered to be unsafe to exercise (e.g. unstable angina, individuals with significant falls-risk) were also considered to be ineligible.

Potential participants were approached during PR classes by CB, a PhD student who was not part of the clinical service and had no prior relationship with the participants. Information about the research study was presented to the group, including the use of a promotional video of walking football recorded with PPIE members. Those who expressed an interest in taking part were contacted upon completion (or discontinuation) of PR and arrangements were made to attend an initial assessment.

### Intervention

The walking football intervention was offered to all participants once weekly, for six weeks (Table 1). Sessions lasted two hours and consisted of one hour of fun warm up games, practice drills and small-sided football matches and one hour of socialising with refreshments. A walking football coach led each session, with assistance from two to three members of the research team (CB + KJL/SLH who both had previous research experience with, and experience supporting, people living with chronic breathlessness). Ten to 12 PPIE members attended each walking football session, in addition to research participants, as peer-support facilitators, facilitating the transition from PR and supporting delivery of the walking football games/drills. The walking football coach and all three members of the research team completed an emergency first aid course prior.

**Table 1.** Walking football for chronic breathlessness - TIDieR checklist.

Item	Description
Brief Name	Walking Football for Chronic Breathlessness
Why	Walking football for people with chronic breathlessness to maintain exercise post-pulmonary rehabilitation (PR). The intervention was developed with PPIE members who had lived experience of chronic breathlessness, health care professionals involved in the delivery of PR and walking football stakeholders (coach and players). PPIE members shaped the intervention design, alongside contributions from frameworks/theory (Behaviour Change Wheel/Capacity OpportunityMotivation-Behaviour), observations of PR and available evidence.
What	Walking football designed exclusively for people with chronic breathlessness, delivered post-PR as a form of exercise-maintenance. 2-hour weekly sessions consisted of practice drills and games, warm-up and cool downs, and small-sided football matches. Other sports such as walking cricket or hockey were also included on occasion to add variety.
Who Provided	Walking football was delivered by an experienced walking football coach, with assistance from the research team. The walking football coach and research team were emergency first aid trained and two were qualified physiotherapists. A minimum of two people oversaw each session to provide additional support for participants who were lower level and to reduce time spent organising equipment (cones, goalposts etc.) between drills/games.
How	Recruitment was conducted in-person at PR programmes. Each PR class typically hosted 4-16 patients. Walking football participation was introduced at the beginning or end of PR.
Where	Walking football sessions were held in a local in-door sports hall with good parking facilities, no steps/stairs and toilets on the ground floor close to the sports hall.
When and how much	After a patient has finished (or discontinued) PR, they were offered the opportunity to play walking football during weekly 2-hour sessions. The study period was for 6-weeks, although participants were free to continue beyond this interest/capacity permitting.
Tailoring	Overseeing walking football required an element of improvisation from the coach, as the footballing ability of participants differed considerably between individuals and even week-to-week due to the presence of fluctuating symptoms. Football drills were tailored to the individual to ensure everyone could partake at an appropriate level of challenge and enjoyability (e.g. a football-drill which requires quick movements may need to be altered to a slower one to avoid over-exertion/injury). Chairs were positioned around the exterior of the pitch allowing for easy substitutions to be made. Frequent rest times were planned into sessions approximately every 15-20 minutes.
Modifications	Walking football was adapted over time to improve safety and enjoyability. Specifically, walking football is self-paced so participants can move at a speed that is comfortable for them, however, it was found that as they grew in confidence, participants became more likely to move too quickly/suddenly, beyond their limitations and at the detriment of their balance, putting themselves and others at a greater risk of falls. The pace of gameplay was carefully controlled and a no-contact rule was applied.
How well	A future definitive trial is planned, potentially using alternative recruitment pathways such as primary care and self-referral, to evaluate the effectiveness of the walking football for people with chronic breathlessness

Patient and public involvement and engagement (PPIE); pulmonary rehabilitation (PR)

### Data collection

Participant characteristics were recorded including; sex, age, height, weight, smoking status, oxygen usage, walking aid usage, underlying cause(s) of breathlessness and co-morbidities. Dyspnoea was assessed via the Modified Medical Research Council Dyspnoea Scale (mMRC).^11^

Data were collected to address the three main objectives: 1. Recruitment rates, adherence, and safety outcomes (Objective 1), 2. Observations and interviews (Objective 2), 3. Physical demand and benefits (Objective 3).

#### Feasibility: Recruitment rates, adherence and safety outcomes

Recruitment rates: The proportion of eligible participants who expressed interest in the study, by providing their contact details for future follow-up at the end of PR, was recorded alongside the proportion of individuals recruited to the study (reported as a percentage of those who initially expressed an interest in taking part). Reasons for not being interested were recorded at a group level during PR sessions and at an individual level for those who initially expressed an interest but later chose not to participate.

Adherence rates: The proportion of participants who completed at least four of six scheduled sessions. The proportion of participants who completed a baseline and post-intervention assessment is also reported.

Safety outcomes: The number of adverse and serious adverse events is reported.

#### Experience and engagement: Observations and interviews

An ethnographic approach was used, consisting of observations carried out before, during and after six walking football sessions by one researcher (BH – a male post-graduate psychology student and yoga instructor). The purpose of the observations was to understand participants’ experiences of walking football and to gain deeper insight into why the activity resonated with some individuals. Reflexivity was supported by the research team consisting of a psychologist (GM), 2 physiotherapists (SLH, KJL), a PhD student (CB) and a medical sociologist (TR) to ensure critical awareness of contextual factors on data collection and interpretation. The researcher was an active observer, taking part in the walking football sessions and engaging in informal conversations. Photographs were taken to support analysis. Detailed field notes were recorded immediately following the session.

Semi-structured interviews were conducted in-person by CB (male – PhD student with a background in psychology) at Teesside University, within two weeks of completing the walking football intervention. The purpose of the interviews was to understand participants’ perspectives on walking football, including any physical, psychological, and social benefits gained through participation. Specifically, we were interested in what made walking football effective in supporting continued exercise after PR, and the factors that might limit its success for others living with chronic breathlessness (Appendix 1). In addition, a PPIE member (female, age 67y), not involved in the co-design of the walking football intervention, conducted an interview with the walking sports coach with support and training offered by the PPIE lead (SS). The purpose of this interview was to understand the experience of walking football for people with chronic breathlessness from the perspective of someone involved in its delivery. Interviews were audio-recorded and transcribed verbatim.

#### Potential benefits: Physical demand and benefits

The following measures were observed during one two-hour walking football session. A participant’s first session was avoided. Cardiovascular demand (heart rate (HR) and distance covered (km)) was measured via a wristband sensor (Polar A370). HR data was categorised by Mean and Peak HR and expressed as a percentage of Estimated HR Max (220 – Age).^12^ The recommended threshold for improving cardiovascular fitness is a mean heart rate of 65%.^13^ Perceived Exertion (Borg Rating of Perceived Exertion (RPE))^14^ and dyspnoea (Modified Borg Dyspnoea Scale (MBS))^15,16^ was also recorded.

The following health-related outcomes were measured at baseline (pre-intervention) and at six weeks (post-intervention): Functional Exercise Capacity (Six-minute walk test (6MWT)),^17^ lower limb strength (30-Second Sit to Stand Test (30-STS)).^18,19^ balance (Mini-Balance Evaluation Systems Test (Mini-BESTest)),^20^ balance confidence (Activities-specific Balance Confidence (ABC) scale),^21^ Health Related Quality of Life (HRQoL) (EQ-5D-5L),^22^ symptom burden (COPD Assessment Test (CAT)),^23^ and psychological status (Generalised Anxiety Disorder (GAD)-7,^24^ Patient Health Questionnaire-9 (PHQ-9).^25^

### Analysis

Numerical data is presented descriptively as frequencies (n) and proportions (%). Field notes and interview transcripts were analysed separately using thematic-template analysis,^26–28^ with a hybrid inductive–deductive strategy before being triangulated and merged for presentation. Neither inductive nor deductive coding was prioritised; instead, the analysis focused on capturing data that were meaningful and useful in relation to the research objectives, whether expressed at a semantic or latent level. The interpretive stance was suspicious-leaning, drawing on contextual knowledge from earlier stages of intervention development and, given the proof-of-concept nature of the study, incorporating relevant behavioural theory (Capacity, Opportunity, Motivation-Behaviour (COM-B)) to inform interpretation.^29^ Despite this theoretical awareness, coding proceeded with a meaning-first orientation, ensuring that participants’ accounts were foregrounded and those data judged to be particularly interesting or relevant were given analytic priority.

## Results

### Study participants

Six participants were recruited (5 male; mean (SD) age 71y (±6.28)) and all had a primary diagnosis of COPD (Table 2). Participants commenced WF within 2 weeks of being discharged from PR.

**Table 2.** Participant demographics.

Sample characteristics	*n*	*%*	*M*	*SD*
Gender				
Male	5	83.33	-	-
Female	1	16.67	-	-
Age (years)	-	-	71.33	6.28
Height (cm)	-	-	164.17	12.86
Weight (kg)	-	-	75.00	12.47
Currently Smoking?	0/6	0	-	-
Smoked Previously?	5/6	83.33	-	-
Smoking Pack Years	-	-	23.00	16.38
Oxygen Therapy Status	0/6	0	-	-
Use of Walking Aid	2/6	33.33	-	-
Primary Cause(s) of Breathlessness				
COPD	6/6	100	-	-
Dyspnoea Severity (mMRC; 0-4 points)	-	-	1.83	1.17 (range 1-4)
Co-Morbidities*	Osteoarthritis, Gout, Hypertension, Vertigo, Epilepsy, Heart Failure, CABG, Aortic Stenosis, LVSD & Severe AS, Diabetes, Stroke, Myoclonic Jerk Median (IQR) number of co-morbidities = 2 (2)			

*To preserve confidentiality, the proportion of participants with each co-morbidity is not reported.

#### Recruitment rates, adherence and safety outcomes

The proportion of eligible individuals who expressed an interest in taking part in the study was 23% (n=36/160). The proportion (%) of those who expressed an interest and consented to participate was 17% (n=6/36). The primary reasons for not expressing an interest were captured across the 27 PR sessions visited at a group level, with the most prevalent reason being a combination of the distance to the sports hall being too far and being unfamiliar with the venue location (noted across 22 of the 27 sessions visited). Following an expression of interest, the primary reasons for non-participation were an inability to contact individuals (33%) and continued enrolment in PR beyond the 6–7-week programme window, which extended beyond the recruitment period (14%). Additional details are provided in Figure 1. Five (83%) of participants completed at least four of six scheduled sessions, and all participants provided a complete data set. One minor adverse event was recorded (a petit mal seizure), which was expected based on past medical history and unrelated to the intervention.

**Figure 1.**
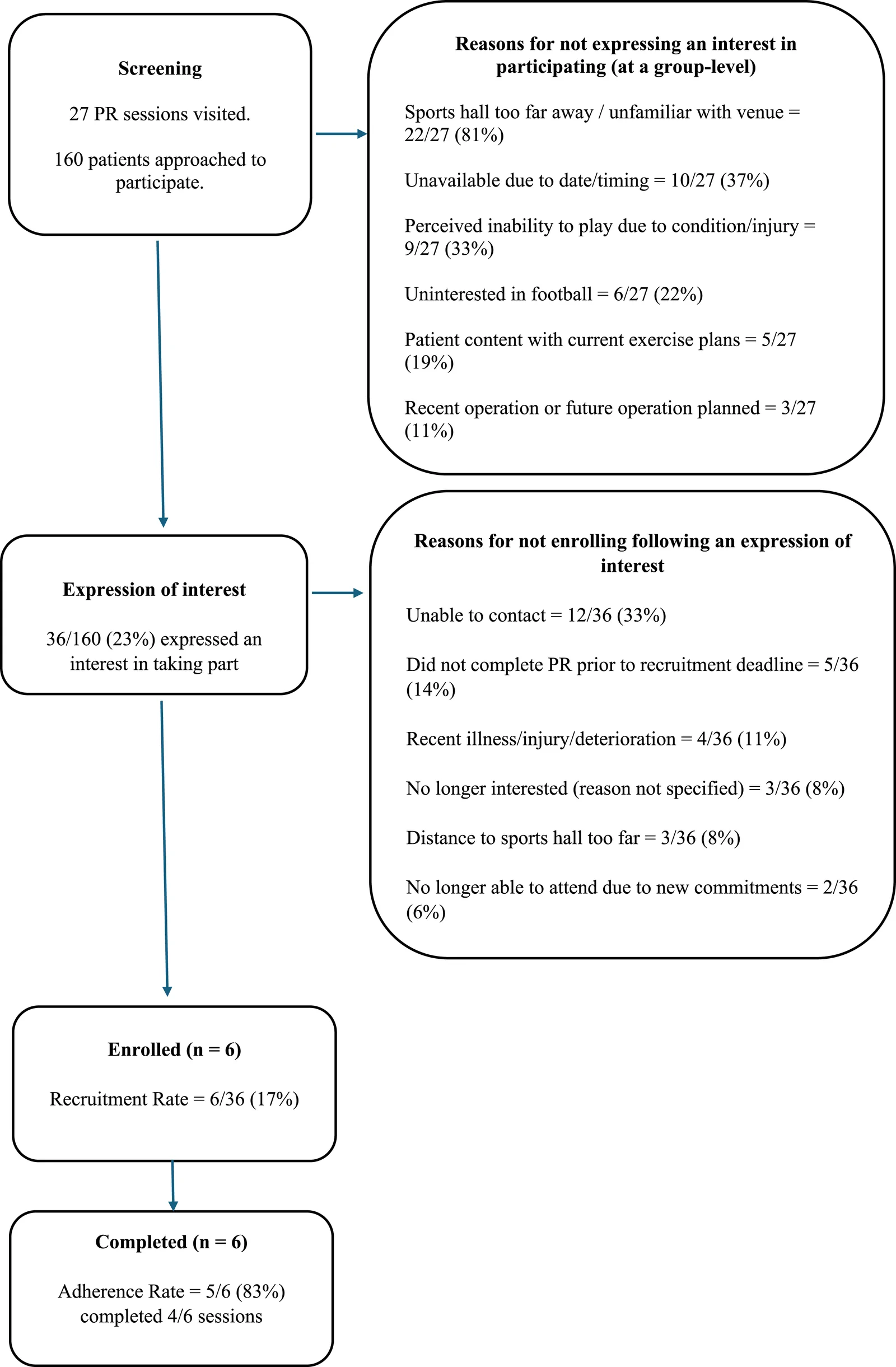
Recruitment flow diagram.

#### Experience and engagement; observations and interviews

Approximately 18 hours of observations were conducted and all six participants completed interviews at 6 weeks (post-intervention), with interview length ranging from 27 to 56 minutes. The interview with the walking football coach was approximately 1 hour and 25 minutes.

Themes are described briefly with supporting extracts from field notes and quotations displayed in Table 3 and mapped to the COM-B model. The completed template consists of six themes, which have been itemised into three higher-order categories. Categories represent the narrative of the study and intervention, namely: *Suitability* (Themes 1 and 2); *Acceptability* (Themes 3 and 4); and *Sustainability* (Themes 5 and 6).

**Table 3.** Extracts from field notes and quotes from interviews mapped to the COM-B model.

Categories	Themes	Example extracts from field notes and quotes	COM-B
Suitability	Capacity	- *Drill is amended for one participant who is using crutches by placing bean bag on his head. This participant made the adjustment spontaneously (FN)* - PR002 s*tates to me “I’m going to sit down a minute” as he leaves an activity, during a drill, to sit in the rest areas by the side of the pitch. I ask if he is ok and he says “Yeah, I just need a little break”. He does appear to be relatively relaxed and in control. He re-joins the drill after a couple of minutes (FN)* - *“I mean we’re all lucky, we’ve all got breathing difficulties, but some are more severe than others.” (Int. PR003)* - *You’ve got to make sure people can get there, car parking spaces. I think that’s very vital… Teesside Uni for example has an amazing sports hall but it’s rubbish for parking.” (Int. COA001)*	Physical Capability; Physical Opportunity
	Beliefs	- PR005: *After missing on her first couple of attempts she scores a goal and states “I’ve found the rhythm now!”(FN)* - *It appeared that PR004 developed confidence throughout the session and/or reduced anxiety as he is less distracted during drills, cessation of self-depreciating commentary and removes hands from pockets (FN)* - *“Well I found it very, very good. And it’s been very helpful to me. Give me something that I thought I would never do and I have done. I just appreciate everything you’ve done” (Int. PR001)*. - *“When I first started this, I was like, They’re not going to be able to do what I want them to do football training wise. But when I’ve seen them do certain activities, I’m so impressed.” (Int. COA001)*	Reflective Motivation; Psychological Capability
Acceptability	Exclusivity	- “*Well I think if it was just a general walking football group, you wouldn’t meet the people who need it. You might get a lot of pensioners that are quite fit and able, who come along and take part and then the ones who require that little bit of a kick – a bit of a start – they just won’t go.” (Int. PR006)* - *“It’s totally different to if you brought a normal guy in to do walking football. To bring one of you guys in to do normal walking football would not work, 100%. I realised that pretty quick” (Int. COA001).* - *“I’ve never thought about getting injured at all. It’s well organised and there’s what, usually three- At least two of you looking after us all the time. No, it’s never crossed my mind that anything could happen to be honest.” (Int. PR002)* - *“There is no pressure for anyone to take part, there is seating there, you can stop when you want, you can start when you want.” (Int. PR006)*	Physical Opportunity; Social Opportunity; Physical Capability
	Enjoyability	- *“We all want to win and score more goals than anyone else, but, no, you don’t have a winner or loser. When he blows the whistle. That’s it, we all have a laugh.” (Int. PR004)* - *PR001: Gives a side on hug after a drill is completed offering physical social support and reassurance. (FN)* - *“I really do enjoy it, I love it, love it, hope it continues” (Int. PR005)* - *“But, even with [PR] there wasn’t really the- They were individual things, you weren’t doing things- There wasn’t a team aspect of it, if you know what I mean? And there wasn’t the opportunity to get together afterwards and have a chinwag. So, no, I think I prefer the football, yes”. (Int. PR002)* - *“You don’t realise what you’re doing. I think if you counted the number of times- Like if you counted the steps you took, or the number of times you did something with your arms, you’d probably find you’re doing more in those couple of hours than you did at [PR].” (Int. PR002)*	Automatic Motivation; Social Opportunity
Sustainability	Benefits	- *Physical positions when kicking ball, one leg, leaning backwards, do not appear to be recognised by the participants. (FN)* - *PR001 and PR003: Take practice swings with tennis rackets between games/drills. Examples of voluntary additional exercise. This is observed by some of the other participants. (FN)* - *Yes, I really enjoyed it. I enjoyed some of the chat with the lads and that. And [PPIE group member], yes, I’m quite good friend with [Him] I don’t know why. He seems to be like a mother hen to me, you know what I mean? (Int. PR001)* - *Well you come away from football, and all your lungs and that are open. Then you go back and you do a few jobs as well. Because you feel alright, but the following day you’re knackered.” (Int. PR004)*	Reflective Motivation; Automatic Motivation; Social Opportunity
	Accountability	- PR001*: “Not feeling so good” almost called in sick “I had the number on the screen” -referring to a coach’s phone number. Speaks about “doing too much” with splash (pulmonary rehab) and WF. “…but I don’t like to let people down”.* (FN) - PR004: *During drill: “I don’t want to let the team down”, “I’m a useless thrower… I have clawed fingers you see.” (FN)* - *“When you coach normally, you’re with a bunch of people who you know but you don’t normally care about them, not the way we care about you guys. It’s hard to explain. After everything we’ve done, I don’t want to walk away.” (Int. COA001)* - *PR001: “Tell (name) I’ll see him soon” before leaving at the end of session (FN)* - PR001, PR002, PR003: *In a tennis style activity, the participants make efforts to include everyone by passing the ball across the court even if there is an available space to score. The coach did not provide any specific instructions for this activity other than to “do” the tennis activity. It seems to have become more of a collaborative passing/rally practice rather than a competitive game although winning shots do receive congratulations and acknowledgement. (FN)*	Social Opportunity; Reflective Motivation

Field notes (FN); Participant (PR); COA (coach); Int (interview quote).

#### Category 1: Suitability – who is walking football suitable for?

##### Theme 1: Capacity

Suitability depended on having the exercise capacity, ability to travel, and sufficient free time to participate. Participants recognised that some people with chronic breathlessness may lack the strength, balance, or endurance required to find walking football safe or enjoyable. Continual adaptations (e.g. use of a crutch), based on the demands of the activity and how they felt that day were observed and supported by reflections on their fluctuating physical state and the need to manage energy carefully. Travel was not an issue for those recruited to participate, though reliance on private transport limited recruitment. Timing was also important; retired participants preferred midday sessions and acknowledged that those working typical hours would require a different schedule.

##### Theme 2: Beliefs in the benefit of activity

Participants strongly believed in the value of continued exercise despite breathlessness, describing it as essential for maintaining health. These beliefs helped them overcome initial doubts and contributed to a growing sense of mastery over their condition. Participants and the coach emphasised understanding personal limits to avoid overexertion, demonstrating both present-moment awareness and future forecasting of symptoms. Participants frequently observed that they could do more than expected once they engaged in the sessions, as they recalled past skills and experiences, drawing out a sense of nostalgia.

#### Category 2: Acceptability – why was the intervention accepted?

##### Theme 3: Exclusivity – designing for chronic breathlessness

Participants emphasised that walking football needed to be designed specifically for people with chronic breathlessness; they would not have joined mainstream programmes. Tailoring created a safe environment where participants could adapt movements, take breaks, and modify equipment. Recruiting only from PR ensured a baseline level of physical ability, supporting psychological safety and social cohesion. Adaptations such as abundant seating, flexible rest breaks, a soft ball, and trained staff further increased confidence and enabled participants to rediscover or learn new physical skills.

##### Theme 4: Enjoyability – prioritising positive experience

Enjoyment was central to acceptability. Sessions were self-paced, social, and varied, helping participants view walking football as something they valued. The balance between competitiveness and inclusivity fostered social connections, namely camaraderie and accountability. Participants often discovered new abilities or rediscovered old sporting skills, (as described in category 1, theme 2) and the enjoyable, game-based format distracted them from breathlessness.

#### Category 3: Sustainability – why did participants sustain attendance?

##### Theme 5: Benefits

Participants described physical, psychological, and especially social benefits; including improved symptoms, increased confidence, and the ability to exceed perceived limitations. The strongest motivator was the social environment: teamwork, shared goals, and supportive relationships created a sense of purpose. Participants learned from one another about managing their conditions and shared healthy lifestyle advice.

##### Theme 6: Accountability

Participants felt accountable to the coach and each other. This sense of responsibility strengthened motivation and reflected the relational aspects of social connectedness. Family interest and pride reinforced this accountability, while the coach also felt responsible for maintaining a safe, meaningful intervention. Friendly competition sat comfortably alongside cooperation, enhancing engagement and enjoyment. Humour, familiarity, and shared experiences were observed and deepened trust between participants and the coach, contributing to a supportive environment that encouraged sustained involvement.

#### Physical demands and benefits

Participants achieved an average mean heart rate (HR) of 97.33 bpm, corresponding to 66% of their estimated maximum HR, with peak HR reaching 81% of estimated maximum, indicating moderate cardiovascular intensity. On average, participants covered 3.23 km per session. Perceived exertion was rated as “Fairly Light” (mean Borg RPE = 12), while breathlessness was perceived as “Moderate” (Modified Borg Dyspnea = 3) whilst playing walking football.

Health outcomes assessed at baseline (pre-intervention) and at 6-weeks (post-intervention) are shown in Table 4.

**Table 4.** Pre-post walking football health outcomes.

Outcome	Mean (SD)		Mean difference	MCID*	MCID change (%)
	*Pre*	*Post*			
Functional exercise Capacity (6MWT;m)	313.67 (123.34)	347.67 (135.29)	34.00	25	^↑^4/6 (66.67)
					^∼^2/6 (33.33)
Lower Limb Strength (30-STS; no. of reps)	10.83 (2.14)	12.00 (4.52)	1.17	2	^↑^3/6 (50.00)
					^↓^1/6 (16.67)
					^∼^2/6 (33.33)
Balance (Mini-BESTest) (total score)	21.83 (6.49)	23.00 (7.16)	1.17	4	^∼^6/6 (100)
Balance Confidence (ABC Scale; total score)	115.17 (32.26)	117.17 (34.10)	2.00	19	^↑^1/6 (16.67)
					^↓^1/6 (16.67)
					^∼^4/6 (66.67)
HRQoL (EQ-5D-5L)					
Index score	0.62 (0.25)	0.67 (0.21)	0.05	0.07	^↑^4/6 (66.67)
					^↓^1/6 (16.67)
					^∼^1/6 (16.67)
VAS Score	71.67 (15.71)	71.67 (22.51)	0.00	8	^↑^2/6 (33.33)
					^↓^1/6 (16.67)
					^∼^3/6 (50.00)
Symptom Burden (CAT; total score)	23 (10.86)	23 (10.02)	0.00	2	^↑^3/6 (50.00)
					^↓^3/6 (50.00)
Anxiety (GAD-7; total score)	10 (8.76)	10 (8.67)	0.00	2	^↑^2/6 (33.33)
					^↓^1/6 (16.67)
					^∼^3/6 (50.00)
Depression (PHQ-9; total score)	10.83 (8.57)	9.67 (9.40)	-1.17	5	^↑^2/6 (33.33)
					^↓^1/6 (16.67)
					^∼^3/6 (50.00)

*Minimal Clinically Important Difference.

^↑^No. of participants who recorded a MCID in a positive direction.

^↓^No. of participants who recorded a MCID in a negative direction.

^∼^No. of participants who did not record a MCID in either direction.

## Discussion

This mixed-methods proof-of-concept study examined the feasibility, acceptability, and preliminary health impacts of a six-week tailored walking football intervention as an exercise-maintenance option following PR. This is the first study to adapt walking football specifically for people living with chronic breathlessness. Overall, the findings indicate that walking football is a promising, engaging, and potentially sustainable form of continued exercise for this population, although significant practical barriers, particularly transport, must be addressed for wider implementation.

Despite high initial interest, recruitment was constrained primarily by reluctance or inability to travel. Participants frequently described not driving, discomfort using public transport, or the burden of long and complex journeys as reasons for declining participation. These barriers are consistent with broader evidence that people with chronic respiratory disease face substantial mobility and accessibility challenges, especially when living in an area with high levels of socioeconomic deprivation.^30,31^ It is important to note that this study was conducted in a region of the UK with some of the highest rates of deprivation and in Stockton-on-Tees 1 in 3 people do not own a car.^32^ Another consideration for a future study is that recruitment was conducted by an individual with no prior relationship with participants. Healthcare professionals involved in participants’ clinical care, who have established trust and rapport, may have been more successful in recruitment, as some individuals may have perceived the intervention as risky or unfamiliar. Importantly, all individuals who enrolled completed the full programme, and five of the six participants subsequently joined a local charity supporting people with chronic breathlessness (Breathe Easy Darlington). This was an unexpected benefit of PPIE and involving members as peer-support facilitators and was realised by engaging with an existing established group. Whilst some people might have found joining a group who were all familiar a little unnerving, qualitative findings show the prior relationships in the group fostered feeling of social connectedness with participants. The group has continued to deliver walking football beyond the study period after securing £10,000 of National Lottery funding. They are now the only team registered with the Walking Football Association specifically for people with chronic breathlessness and have adopted the name The Lung Legends. This sustained engagement, extending well beyond the formal research period, suggests that when access barriers such as transport are removed, walking football offers some potential as a long-term, community-embedded exercise option.

Qualitative findings provide insight into why walking football may be engaged with. Participants consistently described the sport as absorbing, enjoyable, and socially meaningful. The game format distracted from sensations of breathlessness, while teamwork and friendly competition created a sense of purpose often absent from traditional exercise programmes. Social motivations emerged as the dominant driver of sustained participation, far exceeding initial expectations. This may partly reflect the involvement of highly social PPIE members during intervention development, but it also aligns with broader evidence that people with COPD frequently experience isolation and value opportunities for peer connection.^33,34^

Participants demonstrated notable improvements in functional exercise capacity, exceeding the minimal clinically important difference (MCID), despite having recently completed PR, a period when further gains are typically modest. These improvements likely reflect the substantial volume of activity undertaken, with participants averaging more than 3 km of walking per session and achieving mean heart rates exceeding 65% of estimated maximum. This intensity aligns with thresholds associated with cardiovascular and functional benefits in previous walking football and exercise-training research.^12,35^

Participants repeatedly contrasted walking football with PR, which they described as individualised, repetitive, and overly clinical. Although PR remains the gold standard for improving symptoms and functional capacity, its limited social focus may contribute to poor long-term adherence.^36^ Furthermore, it may offer an alternative mode of exercise which might appeal to a broader range of people, and can be delivered in local communities, potentially improving accessibility. This informed the decision to include those who discontinued PR in recruitment. Future research should also consider those who decline a referral to PR, however, given its strong evidence-base it is important to be mindful not to position the intervention as an alternative to PR. The findings here suggest that PR could benefit from integrating more socially oriented, enjoyable elements.

A key strength of the intervention was its tailored approach. Participants emphasised that they would not have attended a generic community sports session. This highlights the limitations of generic older-adult programmes for people with chronic breathlessness and underscores the need for tailored, inclusive approaches. Broader policy initiatives, such as the UK government’s Major Conditions Strategy,^37^ may therefore require greater nuance if they are to effectively support physical activity among people with chronic breathlessness. This is particularly important given the ongoing shift of care from hospitals to community settings outlined in the NHS Long Term Plan,^7^ which assumes that community-based services will be both accessible and appropriate for those with long-term conditions. Ensuring that such policies account for the specific needs and constraints of this population, including mobility limitations, transport barriers, and the requirement for tailored activities, will be essential for equitable implementation.

Despite positive findings, the study has several limitations. Although qualitative and quantitative data were given equal priority, the small sample restricted the strength of conclusions, and findings should be interpreted cautiously. Convenience sampling resulted in a demographically narrow group, predominantly white, middle-class, and with access to transport and free time, limiting representativeness. Although five of the six participants were men, most PPIE members involved in co-design were women. This discrepancy indicates that the gender imbalance in the final sample is unlikely to reflect lower interest among women. Instead, it is more plausible to attribute this to structural barriers, for example, women, especially those living in deprived areas are less likely to own a car,^38^ aligning with transport being the primary barrier to enrolment. Reasons for not expressing an interest in the study were recorded at a group-level, rather than for each individual approached (n = 160). Recruitment occurred via group presentations at the end of PR sessions, and only some attendees provide detailed feedback. Consequently, reported barriers reflect issues raised within the group discussion and may not reflect the concerns of each individual. Furthermore, this recruitment approach may have impacted recruitment rates as some members of the group may have influenced others. Finally, the semi-structured interviews were conducted by a PhD candidate who was involved in the delivery of the intervention, potentially inhibiting any negative feedback concerning walking football. That said, a broader range of researchers were involved in the analysis, helping to mitigate, at least in part, this potential bias.

This proof-of-concept study provides several important lessons for future research: 1. Recruitment may be enhanced when undertaken by healthcare professionals with whom participants have an established and trusted relationship. 2. The accessibility of venues is a key consideration for participation. For some individuals, provision of taxi transport may be necessary due to limitations in public transport. 3. Providing clear information before attendance, for example photographs of the venue, directions, parking arrangements, and toilet locations, may reduce anxiety about a new setting. 3. Partnering with established community or patient support groups to co-deliver sessions may facilitate engagement and ongoing participation. 3. Delivering a maintenance intervention following PR can be challenging because of individual variation in programme duration for different individuals, depending on personal circumstances. 4. No significant safety concerns were identified.

In addition, future work should examine how PR and maintenance programmes such as walking football can be linked to support seamless transition. Research is also needed to explore how PR could incorporate more social elements to enhance adherence. Crucially, transport emerged as the primary barrier to participation; future interventions should consider providing travel support or offering multiple local venues to ensure equitable access.

To conclude, tailored walking football appeared feasible, enjoyable, and potentially sustainable for maintaining exercise following PR among those who enrolled and attended. However, low uptake and accessibility challenges, particularly travel, limit wider conclusions about feasibility and acceptability. The observed engagement, social benefits, and encouraging functional outcomes suggest that walking football may have promise as a community-based maintenance strategy for people with chronic breathlessness.

## Supplemental material

Supplemental material - Walking football for people with chronic breathlessness: A mixed-methods proof of concept study Supplemental material for Walking football for people with chronic breathlessness: A mixed-methods proof of concept study by Callum Bradford, Kirsti J. Loughran, Ben Harrison, Grant McGeechan, Denis Martin, Sophie Suri Noelle Robertson, Gaynor Williams, Tim Rapley, Samantha L. Harrison in Chronic Respiratory Disease

## Data Availability

Data is available and can be shared upon request.*
