# Supplementary material for: Walking football for people with chronic breathlessness: A mixed-methods proof of concept study
Source: Chron Respir Dis. 2026 Sep 21;23:14799731261489273. doi: 10.1177/14799731261489273 (PMC13731885; doi:10.1177/14799731261489273)
Supplement: Supplementary file 1 [file sj-pdf-1-crd-10.1177_14799731261489273.pdf]

Appendix 1. Interview Schedule

How have you found the walking football so far?

Could you see yourself playing walking football consistently / long-term?

Do you think the chance of injury is worth the risk/benefits of playing?

Did you feel safe whilst playing? Was the possibility of injury on your mind?

Did you feel your breathlessness hindered your enjoyment / ability to play?

Have you experienced any health benefits through taking part?

Physical / Mental / Social?

How do you think the walking football programme could be improved?

Sessions per week?

Length of session – hour long enough?

Transition from PR?

What aspects of the football went well? / Did you particularly enjoy?

Favourite training drill / warm-up?

Social aspects?

Coaching?

How does walking football compare to…

Pulmonary Rehab?

Other forms of exercise you have tried?

Your expectations prior to playing?

Were you happy/comfortable with the measures taken?

e.g. 6MWT, STS, HR Monitors, Questionnaires

If you could alter the rules to any other sport, what would you choose?

e.g. walking netball, rugby?`
